# Obstetric and Gynæcological Studies in Berlin

**Published:** 1911-09-30

**Authors:** D. Wilberforce Smith

**Affiliations:** Registrar Samaritan Free Hospital for Women.


### SPECIAL ARTICLES.

### OBSTETRIC AND GYNÆCOLOGICAL STUDIES IN BERLIN.

By D. WILBERFORCE SMITH, F.R.C.S. Eng., Registrar Samaritan Free Hospital for Women.

In visiting Berlin for purposes of study, it is well to have a very clear idea of what one really wants, as much time may be spent on interesting side issues, just as one finds when consulting an encyclopædia. The particular opportunities afforded in diseases of women lie in the examination, diagnosis, and treatment of the plentiful material of the polyclinics, courses of instruction and practice in cystoscopy, watching gynæcological and obstetric operations, preceded often by clinical lectures on the cases to be operated on, courses of operative gynæcology on the dead subject, and in obstetrics, during certain of the holiday months a special resident course of operative work lasting one month and limited to three or four post-graduates, who perform nearly all the operations themselves, under the supervision of the chief assistant of the clinic. Information with regard to these courses may be had from the medical booksellers, and at the Anglo-American Medical Association, which meets every Saturday evening.

### Some Practical Hints.

It is possible to get along without a knowledge of German, but there is a distinct advantage in being able to speak and understand the language, both in saving time in gathering information as to the opportunities for study, time and place of operations to be performed, in appreciating lectures and the remarks of operators, and in securing greater courtesy and attention from the teachers and assistants with whom one comes in contact. There are very large numbers of English-speaking foreigners taking courses in every conceivable subject, and as many of these, who say they are specialists, appear to be in need of completing an elementary curriculum, it is not to be wondered at that English graduates are confounded with these, and that in fact all foreign doctors are regarded as ignorant of the elements of their profession, much of the teaching being adapted accordingly. With a little German and some knowledge of one's subject, one may contradict this impression and make discrimination possible, while with a personal introduction or two added to these, the utmost advantage may be gained from even a short period of study in Berlin or elsewhere.

Polyclinics are the out-patient departments in connection with the hospitals or with the private institutions belonging to the recognised teachers. Courses of attendance three days a week for a month or more are permitted to post-graduates for £2 to £3 a month, when they may examine the patients, discuss the diagnosis with one or other of the assistants, and see or carry out the treatment prescribed. At the private polyclinics the patients are of a slightly better class and pay small fees, generally by insurance in industrial societies.

### New Methods and Theories.

Here there were many new ideas to be gathered, and the treatment differed in the various polyclinics. The first and most practical advance is the universal use of an examination chair, such as is seen also in Dublin, in which the patient is placed in the lithotomy position, but with raised shoulders, and a pan below into which a douche may escape, while the thorough exposure of the parts and favourable position for a thorough bimanual with either hand, the corresponding elbow being supported on one's knee, present advantages over the left lateral which make one wonder at its survival.

Electric vibration is made use of per vaginam and per abdomen for the absorption or stretching of adhesions, with good results in many cases. Retroversion with adhesions was sometimes treated two or three times a week with a mercury colpeurynter, a kind of de Ribes' bag, placed in the posterior fornix and then partially distended with mercury, the patient lying on the back with the hips raised for about an hour. No discomfort was complained of and restoration was in some cases rendered possible.

Gonorrhœa was treated as with us, in some cases with frequent use of salicylic acid pessaries, and treatment was continued until no gonococci could be found in the cervical canal, even after a period. The ready use of the platinum loop in some of the polyclinics, and the immediate staining and examination of the films, commended itself as a most useful practice, and not difficult when done systematically.

Classes in cystoscopy are held twice a week, commencing with the examination of the phantom or model bladder, and then of the actual patients. When proficiency has been attained, one practises catheterising the ureters.

In the gynæcological clinics, or in-patient departments, which consist similarly of hospital and private institutions, the latter being in fact nursing homes on a large scale with several classes of accommodation according to the payment which can be made, there is little for the visitor to see except the actual operating. The unwillingness to show one the after-treatment and give bedside teaching is peculiar, I am told, to Berlin clinics, but even when one has obtained the entrée to the wards there seems to be very little importance attached to the after-treatment, and except to see some special case the professor at a hospital clinic does not personally visit the bedsides.

### With Professor Bumm at the Charite.

Besides a number of private clinics, the professors of which show the greatest courtesy to visitors, there are two principal gynæcological clinics in Berlin, one at the Charité, a hospital intended principally for the education of military surgeons, where von Bergmann, of international reputation, is Professor of Surgery, and the University Hospital, where Prof. Bier directs the surgical clinic, and Prof. Bumm has been transferred from the Charité, where his brilliant gynæcological and obstetric operations and clinical lectures attracted large numbers of visitors and students. He is, by the way, one of the clearest spoken and easiest lecturers to follow that one has heard.

Most operations are performed by Bumm under spinal anæsthesia, with preliminary scopolamine-morphine narcosis, and in the case of Wertheim hysterectomies and other operations occupying more than fifty minutes, chloroform or mixture is given by an apparatus which graduates the dosage accurately, a minimum quantity being required in this sequence. Swabs are taken at different stages in the operations, and placed in sterile Petri dishes kept in the theatre, to see if cultures can be made. In this way he was able to show conclusively that the septic complications, which often spoilt his Wertheim cases, were due to contamination of the wound from the moment the vagina was opened, and he now carefully sterilises this. Generally speaking, he adopts the abdominal and vaginal routes for operating without prejudice, as compared with the somewhat strong bias in favour of abdominal operations in this country, and the extraordinary preference for vaginal operating shown by Strassmann and Mackenrodt. The presence of blood in the peritoneal cavity was often demonstrated, in cases of ruptured tubal pregnancy, by a hypodermic needle put into the vaginal vault and the iliac fossæ. The Alexander-Adams operation of shortening the round ligaments was a favourite method of treating uncomplicated obstinate retroversion, and ventro-suspension for retroversion with adhesions or prolapse.

### The Obstetric Clinic.

Perhaps the material of most general interest was found in the obstetric clinic of the Charité, where post-graduates, as stated above, may obtain exceptional operative experience for a somewhat high fee (£20) during one or other of the holiday months. In the labour ward were six beds, upon which patients in labour were simultaneously under observation. They were examined at intervals by the midwife or intern on duty, always with sterilised gloves, and any complication noted. For example, one was startled one night by a scream for assistance from an examining midwife, who found the cord prolapsed, and kept the head pressed up till she had summoned theatre sister, anæsthetist, and operators, and the patient, having been transferred to the operating table and anæsthetised, was ready for version to be performed.

Whenever the membranes have ruptured, the fœtal heart is counted at intervals of half an hour and a rising rate treated as a sign for interference, even though the mother's condition be still satisfactory. The German school is quite opposed to prolonged use of forceps under any circumstances whatever, for fear of infection, so that if the cervix be not fully dilated, and delivery is to be expedited, a Bossi's dilator may be employed, followed by forceps (Tarnier's axis-traction being employed in every case), or version. The cervix is not examined visually after delivery, unless the continued escape of bright blood suggests laceration, when it is, of course, sewn up at once.

### Midwifery Cases.

Uncomplicated cases are delivered by the midwife or intern, and the perineum preserved by grasping it during expulsive pains with the thumb and fingers close in front of the tubera ischii, thus promoting complete flexion, and when the parietal eminences pass them the fingers and thumb press the perineal tissues powerfully together behind the eminences (in front of them, relative to the child), and may, if the pain pass off, complete the delivery of the head between the pains, or otherwise save the perineum by pushing the tissues together and forwards during the delivering pain. Cases of lacerated vagina or perineum are sewn up within twelve hours. Hæmaturia after operation is treated by the use of a self-retaining catheter till the urine is clear, and intrauterine douches are not usually used. Babies are resuscitated by rubbing, and the passage of a soft tracheal tube into the larynx to suck out fluid, accompanied if necessary by artificial respiration.

The cases are sent off to the wards as soon as possible after delivery, and the clinical charts record the pulse rate by a line drawn in red below that of the temperature, so that a disproportionate rise of pulse rate, indicated by the lines crossing, is at once noted and the vagina carefully cleansed and examined visually, some lochia withdrawn from the uterus by a glass tube for cultivation, and intrauterine douche of aluminium acetate or alcohol administered, with ice-bags or hot fomentations to the abdomen. If streptococci are found, a polyvalent serum is used at once. If gonococci, prolonged rest in bed. Collargol is sometimes used for pyæmic conditions, by intravenous injection. Patients whose temperature and pulse are normal at the sixth day go out the following day.

Bumm always treats eclampsia by evacuation of the uterus, the cervix being dilated by a de Ribes' bag in most cases, followed if necessary, or as an alternative method, by Bossi's dilator, or in cases where a bag could not be introduced and the condition pressing, by vaginal Cæsarean section, the cervical canal being split longitudinally after pushing up the bladder, and the child extracted by a foot, delivery being accomplished within five minutes of the first incision. Subsequent treatment of these cases consisted in rapid infusion of several pints of saline into the thighs, to dilute the toxins, raising the foot of the bed, administration of chloral per rectum, caffein hypodermically, radiant heat sometimes to encourage free diaphoresis, and, lastly, what is considered most important, constant stimulation of the patient's breathing with wet towels, flicking, and rubbing, the mucus being constantly swabbed out of the pharynx, especially after a convulsion, during which last a rubber wedge is inserted between the teeth. Bumm holds that these patients die of œdema of the respiratory passages and lungs as much as from syncope, hence the energetic measures directed to clearing them.

### Dystocia.

Dystocia is never treated in his clinic by induction of premature labour, but each case treated on its merits when labour commences. Thus forceps, if necessary with the aid of a Bossi's dilator, are used for the milder cases, version for others, pubiotomy for greater degrees of contraction if the cervix be fully dilated, followed by forceps delivery in multiparæ, and left to nature in primiparæ with strong pains. For excessive degrees of contraction Cæsarean section is performed, and if the uterus has possibly become infected by vaginal examinations and long rupture of the membranes, Frank's extra-peritoneal operation is preferred, though complete dilatation is here a necessity, the incision having to be placed entirely below the peritoneal reflection, and the difficulty in recognising and stripping down the bladder, without wounding it may make the operation anything but simple. He does not like the classical trans-peritoneal Cæsarean section unless he is sure of an aseptic uterus.

### The Post-Graduate Classes.

All the operations in this department, except pubiotomy and Cæsarean section, may be performed by the post-graduates, though their technique, even to the smallest details of their washing up and their way of picking up needles, is subjected to the closest supervision and criticism by the Professor's chief assistant. A considerable number of forceps cases, laceration sutures, versions, craniotomies, and introduction of de Ribes' bags thus fall to the lot of each man, while the general treatment of the department, as outlined above, may be fully studied in theatre, wards, laboratories, and even the post-mortem room.

